# Supplementary material for: Splice-Junction-Based Mapping of Alternative Isoforms in the Human Proteome
Source: Cell Rep. Author manuscript; Available in PMC 2020 Jan 15. (PMC6961840; doi:10.1016/j.celrep.2019.11.026)
Supplement: 2 [file NIHMS1546469-supplement-2.zip › DF1/PXD006675/Aorta/Aorta_23_PHF1_GNVCLVSMLGCLPLWSPLLEMEHSPVSFQGER.pdf]

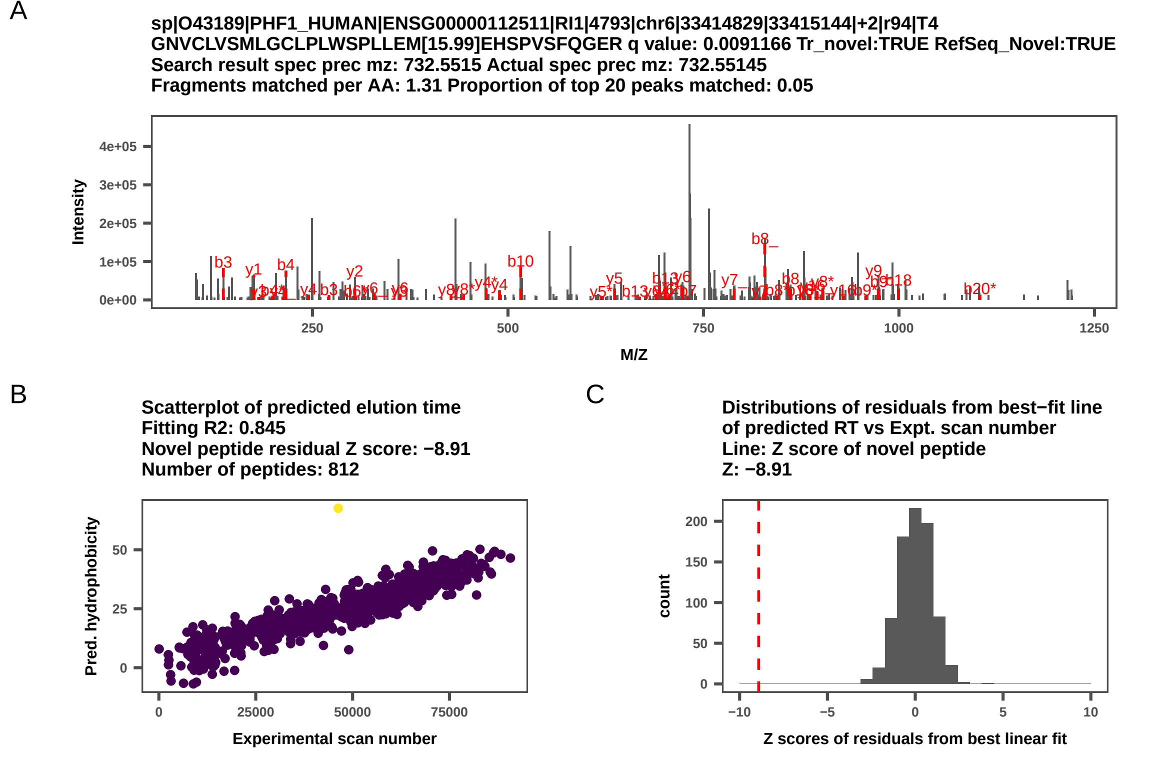
A
sp|O43189|PHF1_HUMAN|ENSG00000112511|RI1|4793|chr6|33414829|33415144|+2|r94|T4
GNVCLVSMLGCLPLWSPLLEM[15.99]EHSPVSFQGER q value: 0.0091166 Tr_novel:TRUE RefSeq_Novel:TRUE
Search result spec prec mz: 732.5515 Actual spec prec mz: 732.55145
Fragments matched per AA: 1.31 Proportion of top 20 peaks matched: 0.05
Intensity
4e+05
3e+05
2e+05
1e+05
0e+00
250
500
750
1000
1250
M/Z
b3
y1
b4
y2
b10
y5
y7
b8_
y9
b20*
B
Scatterplot of predicted elution time
Fitting R2: 0.845
Novel peptide residual Z score: −8.91
Number of peptides: 812
Pred. hydrophobicity
50
25
0
25000
50000
75000
Experimental scan number
C
Distributions of residuals from best−fit line
of predicted RT vs Expt. scan number
Line: Z score of novel peptide
Z: −8.91
count
200
150
100
50
−10
−5
5
10
Z scores of residuals from best linear fit
